# Strengthening provision of essential medicines to women and children in post-Ebola Sierra Leone

**DOI:** 10.7189/jogh.09.010307

**Published:** 2019-06

**Authors:** Rebecca Kahn, Sheriff Bangura, Katrina Hann, Ameet Salvi, Joseph Gassimu, Alpha Kabba, Annelies W Mesman, Kerry L Dierberg, Regan H Marsh

**Affiliations:** 1 Harvard T.H. Chan School of Public Health, Harvard University, Boston, Massachusetts, USA; 2 Partners In Health, Freetown, Sierra Leone; 3 Harvard Medical School, Harvard University, Boston, Massachusetts, USA; 4 Ministry of Health and Sanitation, Freetown, Sierra Leone; 5 New York University School of Medicine, Division of Infectious Diseases, New York, New York, USA; 6 Partners In Health, Boston, Massachusetts, USA

Provision of essential medicines to end users is a critical component of many public health programs and one of six building blocks of the World Health Organization’s (WHO) Health Systems Framework [1]. However, in many countries, the supply chain and pharmaceutical systems remain weak and are costly to improve. We describe a novel quality improvement program for strengthening provision of essential medicines to women and children after the Ebola epidemic in one rural district in Sierra Leone through a public-private partnership between the Ministry of Health and Sanitation (MOHS) and the non-profit organisation, Partners In Health (PIH). To our knowledge, results from a comprehensive, multidisciplinary approach to strengthening supply chain and pharmaceutical systems have not previously been described.

## SETTING

Sierra Leone consistently has one of the highest maternal and child mortality rates in the world, with rates of 1360 deaths per 100 000 live births and 70 deaths per 1000 live births, respectively [2]. A civil war (1991-2002) destroyed much of the health care system, and in 2010, the WHO reported fewer than 150 physicians in a population of over six million [3]. One of fourteen districts in Sierra Leone, Kono has a population of approximately 500 000 [4]. With its large diamond mining industry, Kono district was at the nexus of the civil war, and it remains one of the country’s poorest districts today.

In an effort to reduce maternal and child mortality, in 2010, the MOHS established the Free Health Care Initiative (FHCI), which provides free health care services and essential medicines to pregnant and lactating women and children under five. The Sierra Leone essential medicines list is adapted from the WHO list and undergoes regular review. While the program has been associated with decreases in mortality, drug stock-outs have remained a challenge, impacting its overall success [5]. Limited health care financing hinders the success of the FHCI. More than 60% of health expenditure comes from individual out-of-pocket payments, which can be a barrier to care [6].

Straining the already weak health system, in 2014-2015, Sierra Leone experienced its first known Ebola outbreak, with more than 14 000 cases, nearly 4000 deaths and an estimated 6.85% loss of the health care workforce [7,8], impairing the supply chain and further weakening the FHCI.

PIH began working in Sierra Leone as part of the Ebola response in 2014. In 2015, PIH partnered with the Kono District Health Management Team and leadership from Koidu Government Hospital (KGH) with the goal of improving health care through public health system strengthening. KGH is the only hospital in the district and the only facility in the district capable of performing comprehensive emergency obstetric care, including caesarian sections and blood transfusions [9].

## PROGRAM

Recognizing that reducing maternal and child mortality requires investments in public health systems, including access to essential medicines, KGH leadership and PIH identified successful implementation of the FHCI as a priority towards achieving the shared goals of improving care, increasing patient trust and reducing preventable deaths. To meet this objective, a joint taskforce was developed with representatives from the clinical, pharmaceutical, logistics, infrastructure, and the monitoring and evaluation (M&E) teams, with the goal of understanding the specific failings of the FHCI in reducing maternal and child mortality and how these could be overcome. The taskforce conducted a gap analysis, which involved shadowing staff, studying dispensing practices, conducting inventories, and auditing reporting tools.

The findings revealed that the existing centralized (ie, all drugs supplied from one location for the entire hospital) pharmacy system coupled with low levels of staffing created bottlenecks, resulting in delays for patients to receive medicines. Due to the lack of dedicated pharmacy staff, nurses who were not trained in drug management were responsible for managing medications, diverting their time from patient care. Additionally, while standard operating procedures existed, they were not followed. Lack of documentation and poor quality of storage facilities also led to high rates of drug diversion, normalization of stock-outs of essential pediatric and maternity medicines, and limited information for forecasting, as well as medication spoilage due to inappropriate storage. Combined with insufficient funding and a centralized national push system, these factors impeded patients from receiving essential medicines and undermined trust in the system.

From the gap analysis, we identified four areas for improvement: 1) human resources, 2) drug distribution, 3) storage and inventory management, and 4) information exchange with the national program. To meet the needs identified, we designed a program, implemented from November 2015 to August 2016, to achieve a sustainable, decentralized pharmacy system that could address each of these areas.

### Human resources

One key barrier to success was lack of qualified staff, with only three people dedicated to managing the supply chain and pharmacy at KGH. Together, PIH and the MOHS hired nine additional people to build a pharmaceutical management unit that could effectively support both outpatient and inpatient operations (**Table 1**). PIH staff also supported MOHS staff through capacity building with on the job mentorship and regular trainings on best practices.

**Table 1:** Pre and post program staffing levels and service delivery outcomes

	Pre (Nov 2012 – Aug 2013)	Post (Nov 2015 – Aug 2016)	% increase
**Pharmacy staff**	**No of FTE**		
Pharmacist	1	3	200%
Pharmacy technician	1	7	600%
M&E analysts	0	1	Inf
Logistics staff	1	1	0%
**Total**	**3**	**12**	**300%**
**Patient census**	**No. of patients**		
Pediatric and maternity admissions	2392	3518	47%
Deliveries	418	814	95%

FTE – Full-time equivalent, M&E – Monitoring & Evaluation, Inf – infinity

### Drug distribution

To ensure patients received essential medicines as quickly as possible, we decentralized the storage and dispensing points at KGH by establishing two additional pharmacies on the pediatrics and maternity wards, staffed by the newly hired pharmacy technicians to dispense medicines daily. We also installed emergency cabinets on the wards to provide essential medications and supplies during hours when the pharmacies were closed. The pharmacy technicians maintained stock lists, which the task force evaluated monthly and used for forecasting, allowing the KGH and PIH teams to anticipate needs and procure appropriately.

### Storage and inventory management

The needs assessment identified the quality of the storage facilities as a cause of high rates of stock-outs and product expiration. Medications were stored in a disorganized fashion with minimal inventory tracking, making it difficult for staff to know what products were available. Without a reliable system, needed medications often expired. Additionally, medicines were kept at uncontrolled temperatures, potentially impacting their effectiveness and patient outcomes.

We therefore expanded the hospital warehouse, built shelves, and implemented a stock card system, which improved organization and inventory tracking. PIH also supplemented government provisions of electricity to ensure 24-hour coverage, installed air conditioning units, refrigerators and freezers, and implemented a monitoring system to enhance and maintain temperature control in storage facilities.

### Data collection and communication

The pharmacists and M&E team designed electronic inventory management tools that allowed for near real-time consumption data; these data were used to complete the required forms for the MOHS and therefore enhanced communication about supply needs. We also conducted daily inventories of both the emergency cabinets and pharmacies to prevent stock-outs and improve forecasting. These consumption reports, supplemented by quarterly drug audits, facilitated shared information and increased transparency for all stakeholders.

### Monitoring impact

To evaluate the efficacy of this quality improvement program, we compared key maternal and child health outcomes before and during implementation. Due to the profound disruption of health care delivery during the Ebola outbreak, we chose a period pre-Ebola (November 2012 – August 2013) as the baseline for staffing and patient attendance to compare with the program implementation period (November 2015 – August 2016) using routinely collected program data (**Table 1**). While ideally we would have collected baseline data post-Ebola, record-keeping was limited during the epidemic and immediately afterward, as all actors prioritized the immediate actions required to address critical gaps in the supply chain. During the study time periods, hospital registries were used to collect patient volume data, while pharmacy records were used to collect data on stock-outs.

## PROJECT RESULTS

### Increased patient volume & trust

This program resulted in 100% availability of essential medicines at KGH during the ten-month implementation phase, which has been maintained to date. The increased human resources, decentralized drug distribution systems and robust data collection systems enabled the MOHS to provide approximately 80% of medications, with PIH filling the gap for the remaining 20%. Although stock-outs were frequent prior to program implementation, precise baseline information was not available due to the lack of systems to document and report stock-outs. Patient volume increased substantially from the pre-Ebola baseline to post-project implementation; admissions on the pediatrics and maternity wards increased by 47% and hospital-based deliveries increased by 95%. Community health workers and other community leaders, with whom we had weekly meetings, frequently cited availability of free medicines as one of the main drivers of increased patient satisfaction and trust in the FHCI and KGH. As more than 70% of people in Sierra Leone live on less than one dollar per day, even small medication costs pose a true barrier to accessing care [10].

### Improved quality of care

From reports from clinical staff, availability and timely provision of medicines to patients improved. For example, intravenous artesunate was made available for severe malaria, as well as surgical and anesthesia supplies for the operating theatre, enabling emergency C-sections; previously both were stocked out frequently, severely limiting patient care. Importantly, the presence of pharmacy technicians shifted the distribution burden away from nurses, allowing them to focus primarily on direct patient care and reducing the rates of missed medication doses and preventable deaths.

**Figure:**
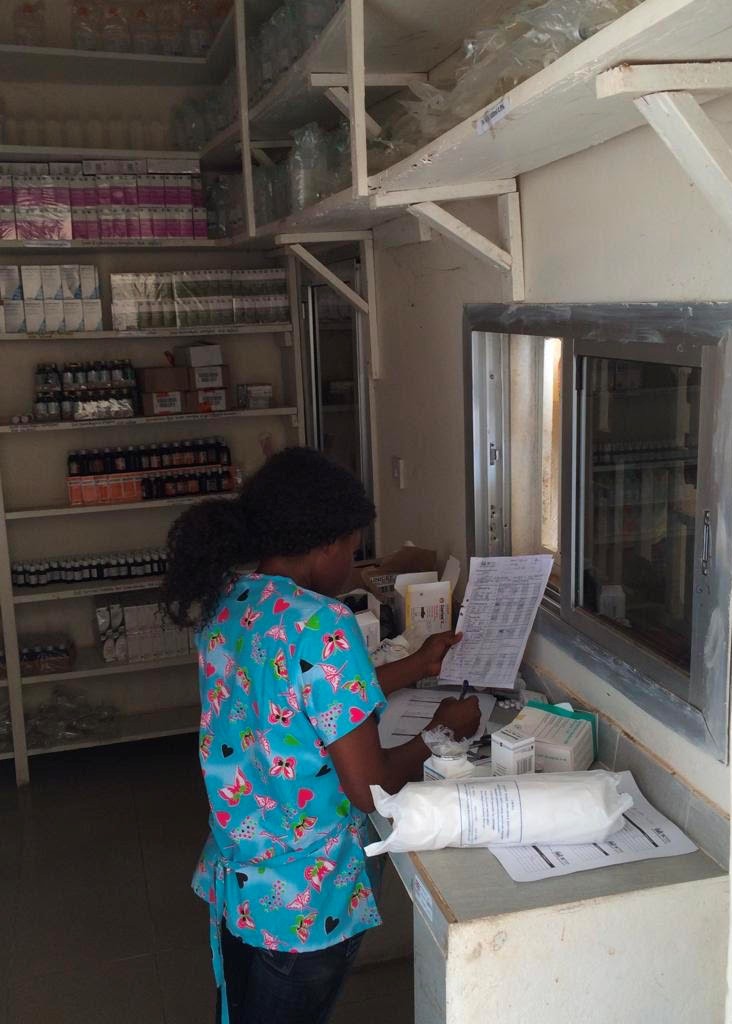
Photo: Pharmacy technician dispensing medicines in the new pediatric pharmacy at Koidu Government Hospital in Kono, Sierra Leone (from the collection of Sheriff Bangura, used with permission).

### Increased transparency and data-driven decisions

The availability of near real-time consumption data increased transparency and facilitated evidence-based forecasting. It allowed PIH to focus on filling in gaps in medication supply while reducing waste from duplicative ordering. Aligning the data tools ensured all MOHS reporting requirements were met, and the data empowered district staff to effectively communicate their needs to the central level. Following program implementation, KGH received an award from the Sierra Leone Anti-Corruption Commission for best “Transparency and Implementation of the FHCI.”

KGH staff also applied lessons from this program to improve forecasting for district-wide programs, such as HIV and malaria. The national programs provide drugs based on historic consumption data. If facilities under report consumption, the district does not receive adequate supply. The taskforce therefore conducted trainings with all health facilities and provided many in-person supervisory visits, resulting in an increase in reported consumption data and reduced stock-outs.

### Costs

This program supplemented KGH’s annual operating costs as part of PIH’s ongoing clinical and logistics support. While a formal cost-effectiveness analysis is outside this report’s scope, we estimate that the additional annual expenditure from PIH was approximately US$150 000, with roughly 10% in one-time infrastructure costs and 90% in recurring operating costs (ie, salaries for additional staff, training, fuel, medicines). While these costs are not insignificant, there were savings that offset these expenditures, including reduced product expiration rates and reduced drug diversion. The estimates also do not capture the impact of improved trust, enhanced patient care and likely reduced mortality rates that resulted from the 100% availability of essential medicines and the creation of a reliable supply chain system. These outcomes, associated with the substantial increase in patient volume in less than one year, indicate the program is a worthwhile and replicable investment at the district level.

## CONCLUSION

The success of our program stemmed from shared ownership between PIH and the MOHS and the multidisciplinary strategy. Our committed partnership worked towards the common goal of improving access to essential medicines through strengthening the MOHS system, rather than creating parallel systems. By developing strategic objectives and empowering people with resources to succeed, we ensured investments were targeted in areas of greatest need and created capacity in the staff.

The increased transparency and data-driven processes improved the relationship between the national and district programs, with Kono now serving as a model district. This program could be replicated in other districts, creating a nationally decentralized pharmacy system, with coordination by the MOHS. Similar investments in human resources, decentralized distribution systems, inventory management, and data systems could strengthen supply chains around the world.

### Limitations

The pharmacy and supply chain work were one part of PIH’s broader health systems approach; therefore, it is not possible to completely isolate the effects of this program on these results. However, abundant anecdotal evidence suggested that the reliable availability of free essential medications significantly influenced these results. In addition, a lack of accurate baseline data on stock-out rates and the inability to control for confounding are limitations. Nonetheless, the strengthened data systems implemented during this project will allow for more accurate data that can be used to draw stronger evidence-based conclusions as the project grows.

### Future directions

Future work will collect additional data regarding costs and the impact on hospital utilization rates and mortality. We also plan to work with the MOHS to assess the feasibility of expanding this project to other districts and to apply lessons learnt to improve access to essential medicines for all patients across Sierra Leone.
